# Venous blood sampling for less invasive in vivo quantification of synaptic density with constant infusion of [^18^F]SynVesT-1 and PET

**DOI:** 10.1186/s13550-025-01200-2

**Published:** 2025-02-03

**Authors:** Ruth H. Asch, Mika Naganawa, Karina Moisieienko, Mia Weed, Michael Kapinos, Ming-Qiang Zheng, Ansel T. Hillmer, Richard E. Carson, Robert H. Pietrzak, Irina Esterlis

**Affiliations:** 1 Department of Psychiatry, Yale School of Medicine, New Haven, CT 06510 USA; 2 Department of Radiology & Biomedical Imaging, Yale School of Medicine, New Haven, CT USA; 3 Department of Biomedical Engineering, Yale School of Engineering & Applied Science, New Haven, CT USA; 4 U.S. Department of Veteran Affairs National Center for Posttraumatic Stress Disorder, Clinical Neurosciences Division, West Haven, CT USA

## Introduction

Positron Emission Tomography (PET) imaging with radiotracers targeting the synaptic vesicle glycoprotein 2 A (SV2A), an integral glycoprotein present in the membrane of all synaptic vesicles throughout the central nervous system [1–4], provides a method for the in vivo quantification of synaptic density [5]. Since the development of the first SV2A radiotracer, [^11^C]UCB-J [6, 7], and later, the fluorinated tracer [^18^F]SynVesT-1 [8, 9], the use of SV2A PET has expanded rapidly due to the broad utility in being able to visualize and quantify synapses in the living human brain, and has proven to be invaluable tools for investigating relationships between synaptic density and brain function within the context of psychiatric [10, 11], neurological, and neurodegenerative disease [12–16], as well as synaptic changes associated with typical development and aging [17, 18]. 

The “gold standard” quantification method applied to PET studies with SV2A radiotracers, including [^18^F]SynVesT-1, is using the one-tissue compartment model (1TCM) to estimate equilibrium parameters [5, 9, 19]. This approach requires at least 60 min of dynamic imaging data collection following administration of the radiotracer as a bolus injection, along with arterial blood sampling in order to measure the input function [5, 9, 19]. However, we recently demonstrated that this relatively long scan duration, which may be a limiting factor for certain individuals or entire populations, can be reduced. By administering [^18^F]SynVesT-1 as a bolus plus continuous infusion (B/I), we were able to reliably achieve a state of equilibrium. Thus only a 30-minute scan (90–120 min post-injection) is required to calculate radiotracer volume of distribution (*V*_T_) as the ratio of the metabolite-corrected concentration of radiotracer in the target tissue to that in arterial plasma at equilibrium (*C*_P,90−120_) [20]. While a shortened scanning period improves study feasibility, the equilibrium method is still reliant on arterial blood sampling for *V*_T_ estimation. Arterial blood sampling is not only technically challenging and subject to errors, it also remains a substantial concern when it comes to study participant comfort and safety, representing a significant reason for study drop-out and data loss [21, 22]. Therefore, the primary goal of the current studies was to test whether less-invasive venous blood sampling can be used in place of an arterial sampling to estimate [^18^F]SynVesT-1 *V*_T_ when using an equilibrium analysis.

## Methods and materials

### Human subjects

Data were analyzed from 19 participants (10 male; 55.6 ± 10.1 years), including nine healthy adults and ten adults with lifetime history of mood disorder diagnosis, seven of whom were currently experiencing a major depressive episode, with the remaining three being in remission from depressive symptoms as determined by the Structured Clinical Interview for DSM Disorders. These subjects were recruited for ongoing studies in an older adult population. As such, three out of the nine healthy participants were taking prescription medications, including psychoactive medications (gabapentin, venlafaxine, paroxetine, semaglutide). Additionally, six of ten participants with lifetime history of mood disorder were taking psychoactive medications. Scanning procedures were approved and overseen by the Yale University Human Investigation Committee and Radiation Safety Committee in accordance with the United States federal policy for the protection of human research subjects. Written informed consent was obtained from all subjects after complete explanation of study procedures and prior to participation.

### Radiotracer injection and data acquisition

[^18^F]SynVesT-1, was synthesized as previously described [8]. Doses of 163.4 ± 27.0 MBq with high molar activity (142.0 ± 83.9 GBq/µmol) were delivered as bolus (1 min) followed by a constant infusion (*K*_bol_=150 min) using an automated infusion pump program (PHD 22/2000, Harvard Apparatus). Imaging data were acquired for one hour from 60 to 120 min following the injection on a Siemens High Resolution Research Tomograph (*n* = 12) or Biograph Vision (*n* = 7) PET camera [20]. 

### Arterial and venous input functions

Radial artery and venous (at the antecubital area) catheters were placed in the arm contralateral to the venous catheter used for administration of [^18^F]SynVesT-1. Manual arterial samples were taken at 60, 75, 90, 105, and 120 min-post injection. Manual venous samples were taken at 60, 90, and 120 min-post injection. Whole blood and plasma activities were measured at all timepoints (Wizard 2480, Perkin Elmer). Radio-HPLC was performed on plasma samples from 60, 90 and 120 min to measure parent fraction [8, 23], and metabolite-corrected *C*_P,90−120_ were computed as the product of parent fraction and plasma concentrations and then linearly interpolated to the PET frame times as previously described [24]. 

### Equilibrium ratio analysis

Images were coregistered to each participant’s T1-weighted MRI images using a six-parameter mutual information algorithm (FLIRT; FSL version 3.2), which was then coregistered to the MRI template by nonlinear transformation using Bioimagesuite (version 2.5). Time-activity-curves (TACs) were extracted from 10 regions of interest (ROIs) delineated using the Anatomical Automatic Labeling atlas [25], specifically frontal, parietal, temporal, and occipital cortices; caudate, putamen, thalamus, hippocampus, amygdala, and cerebellum, as well as a TAC for a gray matter mask applied to the whole brain (“whole brain GM”). A TAC was also generated for a 2 mL volume of white matter within the centrum semiovale as previously described [26] and detailed in the Supplement. Regional *V*_T_’s were estimated as the ratio of the radiotracer concentration in the target tissue region ( *C*_T_ ) to that in arterial (*V*_T, art_) or venous plasma (*V*_T, vein_) at equilibrium. We previously determined equilibrium is achieved in arterial blood 90–120 min into the [^18^F]SynVesT-1 infusion [20]. Here, we evaluate the quality of achieved equilibrium in arterial and venous plasma and of regional *V*_T_ values (i.e., *C*_T_/*C*_P_ ratios) as the slope of the line fit between 90 and 120 min [20]. 

### Statistical analyses

All tests were two-tailed with a significance threshold of ⍺=0.05. IBM SPSS Statistics was used for data analysis (version 29.0.1.0) and GraphPad Prism (version 10.3.0) was used for data visualization. Data are presented as the mean ± standard deviation (SD). Coefficient of variation (COV) is expressed as:

$$\:\left(\frac{\sigma\:}{\mu\:}\right)\times\:100=\%COV$$

The quality of the equilibrium in arterial versus venous plasma (SUV/hour) was assessed by a paired-sample t-test. Single sample t-tests were used to evaluate whether slopes were significantly different from zero. Differences between venous and arterial measures were calculated as:

$$\:\left[\left(\frac{Venous}{Arterial}\right)-1\right]\times\:100=\%Difference$$

Differences between groups were assessed by independent sample t-tests. After conducting Shapiro-Wilk’s normality test, relationships between two continuous variables were assessed by Pearson’s or Spearman’s correlations as appropriate.

## Results

### Comparison of arterial and venous CP,90−120

**Fig. 1:**
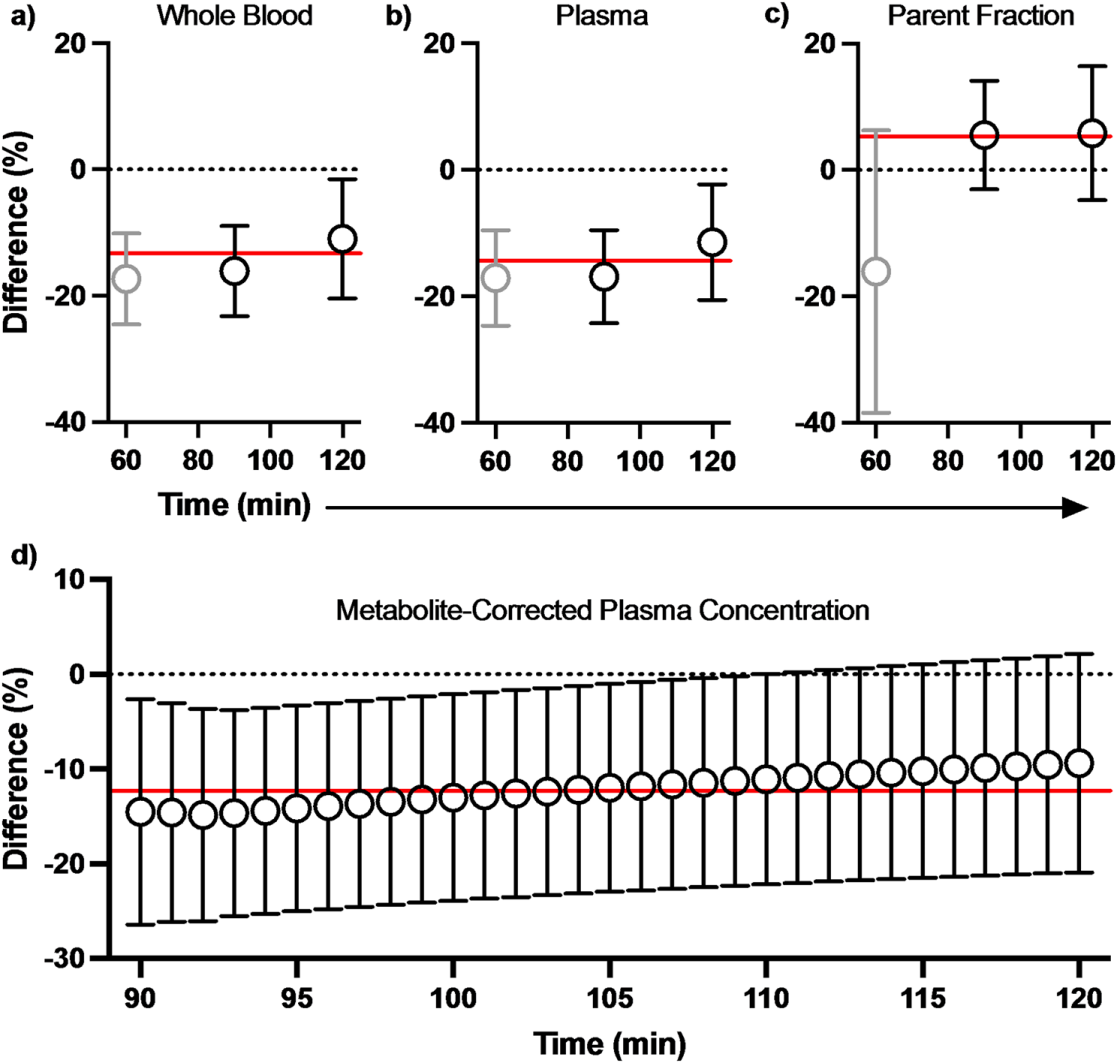
The difference (%) between arterial and venous [^18^F]SynVesT-1 concentrations in the (**a**) whole blood, (**b**) plasma, and (**c**) differences in plasma parent fractions, all as a function of time-post-injection. (**d**) The difference (%) between arterial and venous metabolite-corrected *C*_P,90−120_. Data are presented as the population mean ± SD at each timepoint. The solid horizontal line represents the population mean for the %difference of individual 90–120 min (i.e., at equilibrium) averages

Venous [^18^F]SynVesT-1 concentration at equilibrium (90–120 min) underestimated that which was observed in arterial blood (Fig. 1a; -13.5 ± 7.0%) and plasma (Fig. 1b; -14.2 ± 7.0%). Conversely, the venous plasma parent fraction tended to overestimate arterial values (4.7 ± 8.7%; Fig. 1c). Ultimately, the 90–120-minute venous metabolite corrected *C*_P,90−120_ underestimated arterial *C*_P,90−120_ on average by 12.3 ± 10.6% (Fig. 1d; Table 1).

**Table 1:** Mean and variance of *C*_P,90−120_ and brain region *V*_T_ values, and agreement between arterial and venous-derived values (*n* = 19)

	Arterial		Venous		ICC	Bias (%)
	Mean(SD)	COV	Mean(SD)	COV		Mean(SD)
*C*_P,90−120_ (SUV)	0.32(0.07)	20.92%	0.28(0.07)	24.15%	0.92	-12.27 (10.90)%
Frontal Ctx.	20.99(4.28)	20.49%	24.08(6.11)	25.39%	0.89	15.06 (14.48)%
Parietal Ctx.	20.58(4.08)	19.80%	23.66(5.62)	23.74%	0.88	
Temporal Ctx.	21.89(4.69)	21.42%	25.19(6.59)	26.15%	0.89	
Occipital Ctx.	20.71(4.08)	19.69%	23.79(5.61)	23.58%	0.88	
Hippocampus	14.43(3.23)	22.56%	16.54(4.68)	28.31%	0.90	
Amygdala	18.30(4.35)	23.79%	21.08(6.12)	29.03%	0.91	
Thalamus	15.55(3.40)	21.85%	17.90(4.76)	26.61%	0.90	
Caudate	18.73(4.51)	24.07%	21.60(6.42)	29.69%	0.91	
Putamen	21.96(4.75)	21.62%	25.28(6.62)	26.17%	0.90	
Cerebellum	16.12(3.15)	19.55%	18.52(4.34)	23.45%	0.88	
C. Semiovale	3.35(0.60)	18.02%	3.81(0.56)	14.67%	0.83	
WB GM	19.59(3.97)	20.26%	22.48(5.73)	25.49%	0.88	

Abbreviations: equilibrium metabolite-corrected plasma concentration (*C*_P,90−120_), volume of distribution (*V*_T_), standardized uptake value (SUV), standard deviation (SD), coefficient of variance (COV), intraclass correlation coefficient (ICC), cortex (Ctx), centrum semiovale (C. semiovale), whole brain gray matter mask (WB GM)

**Fig. 2:**
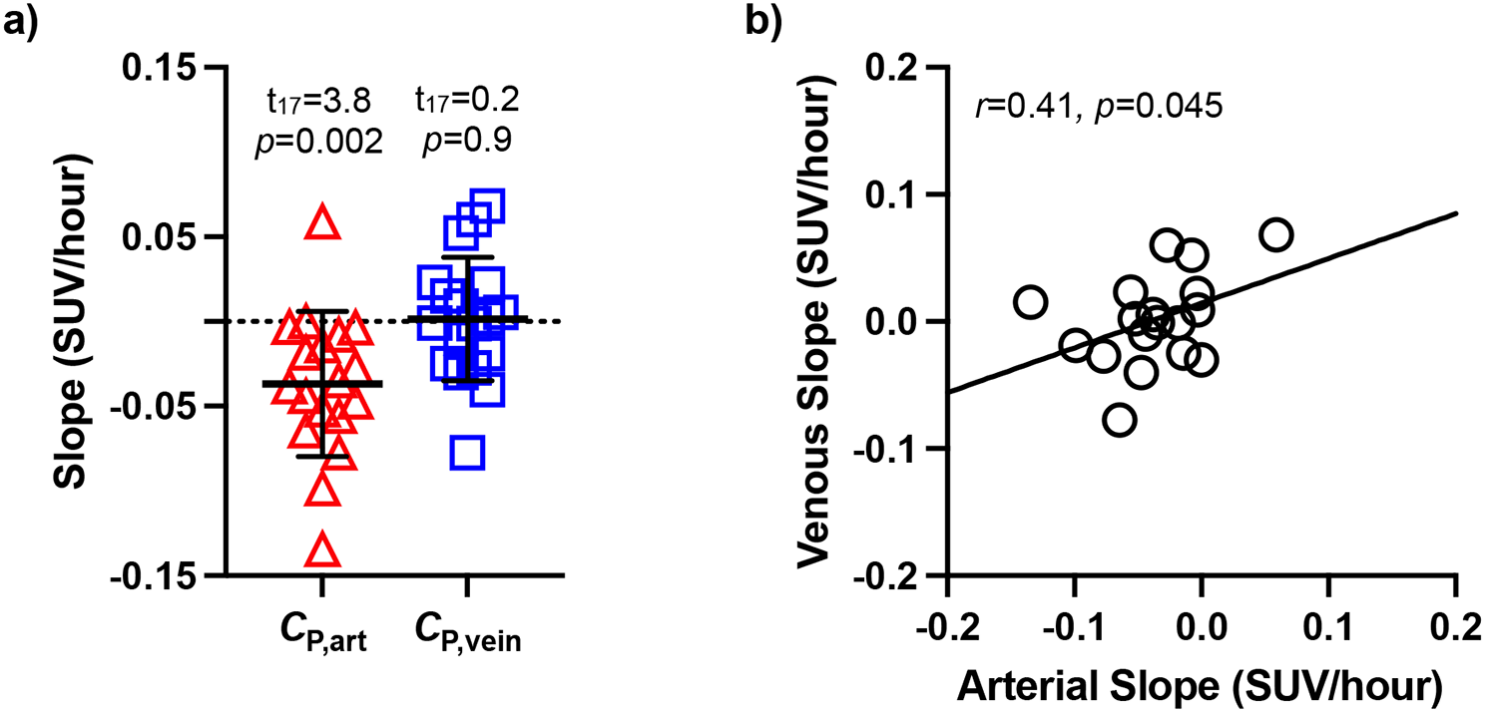
(**a**) Arterial and venous input functions slopes (SUV/hour) at equilibrium (90–120). Data presented as the mean±SD for *n* = 18 individuals. Results of single sample t-tests testing whether slopes are significantly different than zero are provided. (**b**) The correlation between arterial and venous *C*_P,90−120_ slopes

**Fig. 3:**
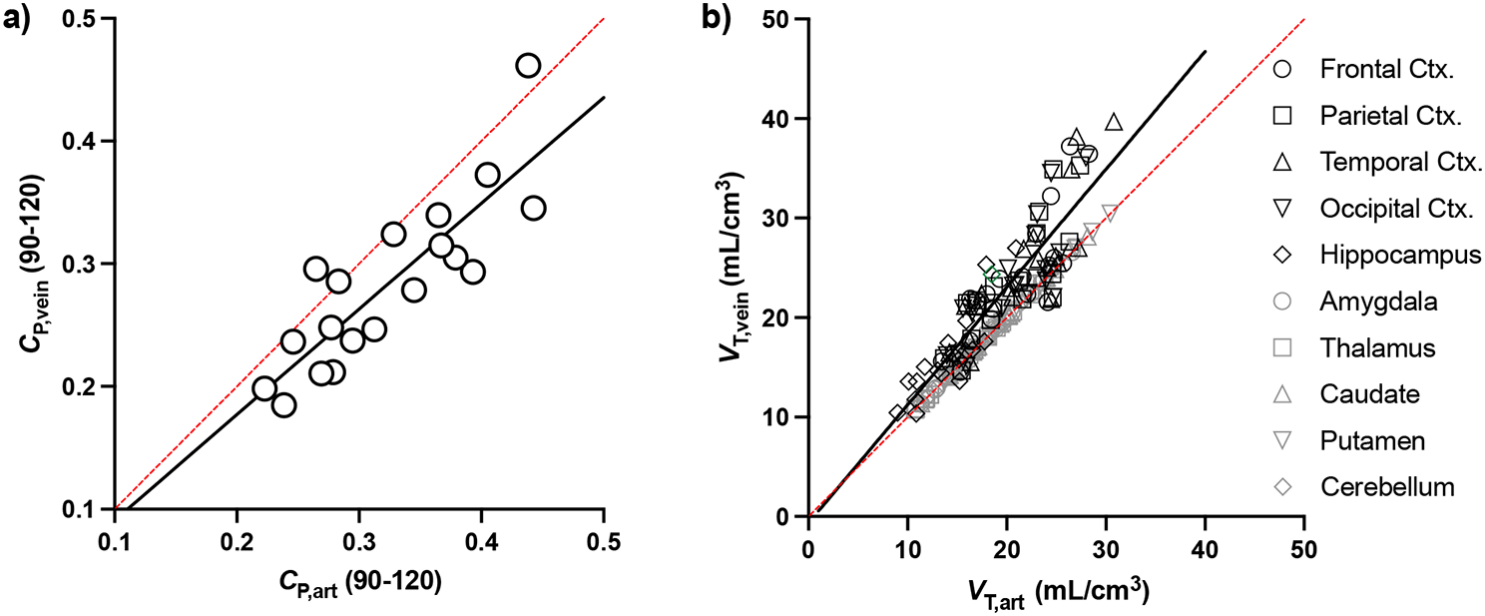
The correlation between (**a**) standardized uptake values (SUV) of equilibrium (90–120 min) arterial (*C*_P, art_) and venous (*C*_P, vein_) plasma (*n* = 19 individuals). (**b**) The correlation between regional volume of distribution (*V*_T,_ mL/cm^3^) estimated using arterial (*V*_T, art_) versus veinous (*V*_T, vein_) equilibrium *C*_P_ (*n* = 19 individuals; *n* = 10 ROIs). The solid lines represent the linear correlation (equation displayed). The dashed lines are the lines of identity

When assessing *C*_P,90−120_, one participant had arterial and venous slopes > 3 SDs beyond the mean and was thus excluded from the slope analysis. For the 18 remaining participants, slopes of arterial input functions were negative for all but one participant and mean absolute value of the slope was − 0.04 ± 0.04 SUV/hour. The venous *C*_P,90−120_ slope was positive on average, with positive slopes observed in half of the subjects (*n* = 9) and a mean absolute slope value of 0.001 ± 0.04 SUV/hour. While arterial *C*_P,90−120_ slopes were significantly larger than venous (Fig. 2a; t_17_ = 3.8, *p* = 0.002), arterial and venous slopes were significantly correlated (Fig. 2b; *r* = 0.41, *p* = 0.045). Despite these differences between arterial and venous equilibrium concentrations, parent fractions, and slopes, the arterial and venous metabolite-corrected *C*_P,90−120_ were highly correlated (Fig. 3a; *r* = 0.852, *p* < 0.001). Similarly, slopes of regional arterial *C*_T_/*C*_P_,_90−120_ ratios were positive on average (whole brain GM: 0.86 ± 2.08 *C*_T_/*C*_P_ per hour), with a mean absolute magnitude of 1.53 ± 1.53 *C*_T_/*C*_P_ per hour (Figure S1). Slopes for venous *C*_T_/*C*_P_,_90−120_ ratios were of a similar absolute magnitude (whole brain GM: 1.86 ± 1.17 *C*_T_/*C*_P_ per hour) but were negative on average (-0.90 ± 2.12 *C*_T_/*C*_P_ per hour).

### Comparison of venous and arterial CP,90−120 and equilibrium VT values

Mean ± SD and COV for arterial and venous *C*_P,90−120_ and *V*_T_ values are presented in Table 1. Regional *V*_T, vein_ values overestimated *V*_T, art_ values by an average of 15.06 ± 14.48%. Gray matter regional *V*_T, vein_ values also tended to be slightly more variable than *V*_T, art_ values, with the COV of the population (*n* = 19) mean ranging from 23.45% (cerebellum) up to 29.69% (caudate), versus 19.55% and 24.07%, respectively, for *V*_Tart_. The linear relationship between *V*_T, art_ and *V*_T, vein_ was significant (Fig. 3b, Figure S2; *r* = 0.899, *p* < 0.001).

### Evaluating the effect of biological, demographic, and injection parameters on bias

**Fig. 4:**
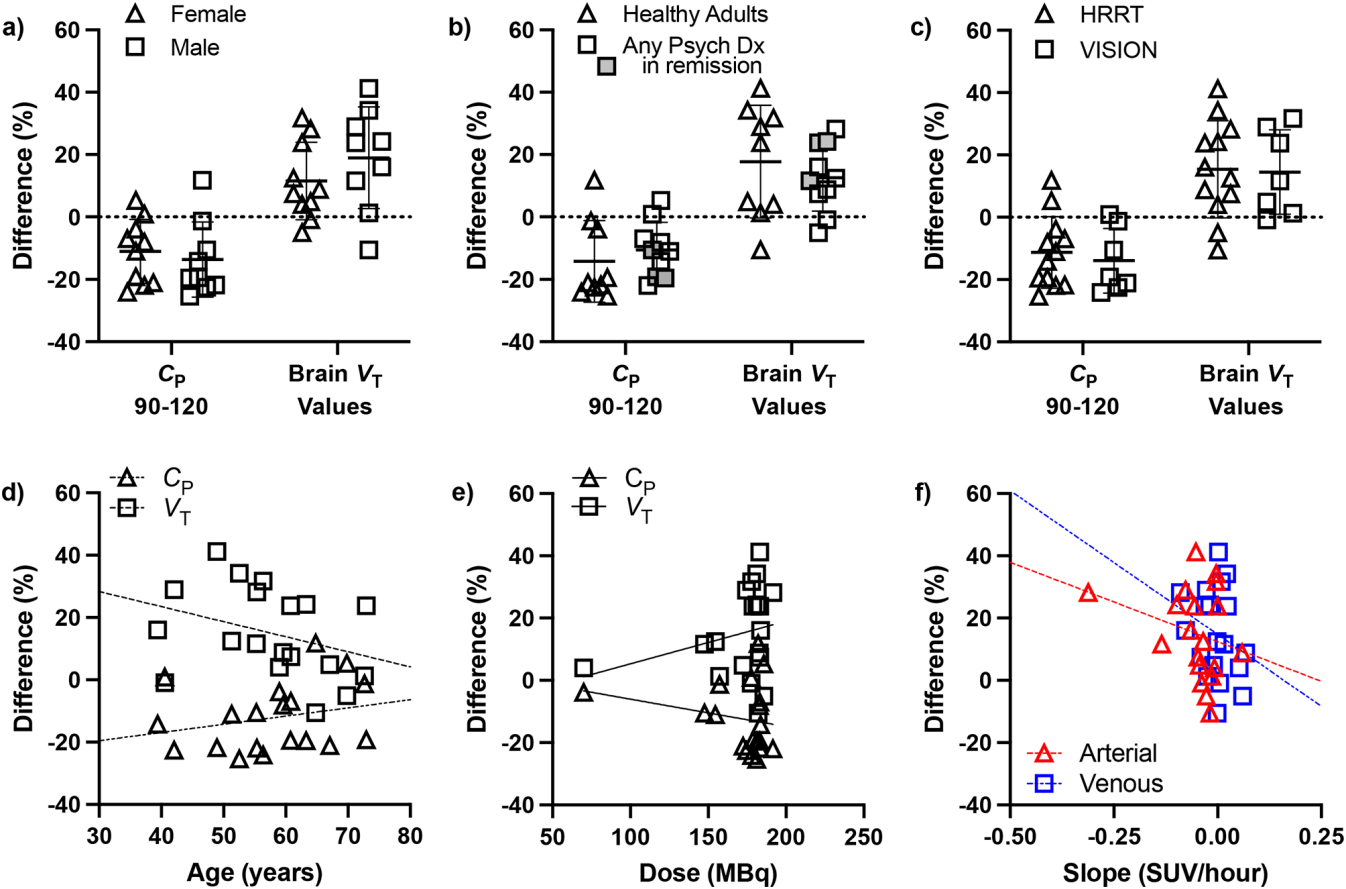
The Influence of (**a**) sex, (**b**) presence of a psychiatric diagnosis, or (**c**) PET camera. Individual values shown with mean ± SD. Correlations between (**d**) age, and (**e**) injected activity on the difference between arterial versus venous input functions and *V*_T, art_ versus *V*_T, vein_ values. (**f**) The influence of the quality of equilibrium (as measured by the slope of *C*_P,90−120_) on the difference between *V*_T, art_ versus *V*_T, vein_ values. Abbreviations: psychiatric diagnosis (Psych Dx)

There was no significant effect of sex (Fig. 4a) Psychiatric diagnosis (Fig. 4b) or scanner (Fig. 4c) on the %difference between *C*_P,90−120_ or *V*_T_ values (all *p* > 0.1). Relationships between %difference and age (Fig. 4d; *C*_P,90−120_: *r* = 0.243, *p* = 0.315; *V*_T_: *r*=-0.337, *p* = 0.159), and [^18^F]SynVesT-1 dose (Fig. 4e; *C*_P,90−120_: ⍴=-0.072, *p*= 0.769; *V*_T_: *r* = 0.255, *p* = 0.292) were not significant. Similarly, the quality of equilibrium achieved was not associated with %difference between *V*_T_ values (Fig. 4f) for arterial (*r*=-0.266, *p* = 0.272) and venous (*r*=-0.263, *p* = 0.277) *C*_P,90−120_.

## Discussion

The purpose of this study was to evaluate whether a venous blood sampling can be used in place of arterial sampling to calculate equilibrium *V*_T_ for [^18^F]SynVesT-1 PET. Indeed, we demonstrate here, in a sample of 19 individuals with and without psychiatric disorders, that *V*_T, vein_ and *V*_T, art_ are highly correlated across individuals and across gray matter brain regions (*r* = 0.899, *p* < 0.001) despite *V*_T, vein_ overestimating *V*_T, art_ by 15% on average.

The present analyses focus on the 90–120 min period, as we previously determined this to be the time at which we reliably achieve a state of equilibrium following B/I delivery of [^18^F]SynVesT-1 [20]. Here we found that equilibrium venous plasma concentrations of [^18^F]SynVesT-1 underestimate arterial plasma concentrations by an average of 14%, and venous parent fraction overestimated arterial values by an average of 5%, resulting in metabolite-corrected venous *C*_P,90−120_ underestimating arterial *C*_P,90−120_ by 12%.

Our evaluation of the *C*_P,90−120_ slopes indicate we are successful at achieving a state of equilibrium in the arterial plasma at 90–120 min, with venous slops being smaller (in absolute value) relative to the arterial slopes and were not significantly greater than zero. Despite these differences, across the 19 subjects tested, venous and arterial *C*_P,90−120_ were highly correlated (*r* = 0.852, *p* < 0.001). Importantly, we demonstrated that observed biases between arterial and venous sampling is not systematically influenced by demographic or clinical characteristics, including sex, age, and psychiatric diagnosis. We also did not detect an effect of the injected activity or the quality of the achieved equilibrium at 90–120 min. Therefore, despite underestimation of the metabolite-corrected *C*_P,90−120_ and overestimation of *V*_T_ values introduced by venous sampling, individual bias does not appear to be influenced by common biological or technical factors and is consistent across individuals. It is important to note that using the venous *C*_P,90−120_ introduced greater variability in the *V*_T_ estimates. However, the modest increase in variability observed and the resulting slight decrease in power would in most cases be outweighed by the reduction in data loss due to technical errors, participant drop-out, and improved recruitment.

This study is not without limitations. First worth noting is the heterogeneity of the sample population, particularly the inclusion of individuals taking medications with the potential of impacting tracer binding, metabolism, and clearance. However, by including these individuals this sample is arguable more representative of the population at large, making the results of these analyses more generalizable. While we did not observe a significant effect of medication status on the *C*_P, art_ vs. *C*_P, vein_ bias at equilibrium (Figure S3), in the future, it is advisable to continue to examine and covary for the potential impact of medication status on desired outcome measures. Second, radiotracer protein binding (plasma free fraction, *f*p) was only measured in arterial samples. We did not observe differences in arterial *f*p as a function of diagnosis or medication status (Figure S4), but we are not able to comment on potential differential impact of diagnosis, medication status, or other factors on venous *f*p, and will therefore need to be evaluated in the future.

In conclusion, using the B/I approach for [^18^F]SynVesT-1 PET to achieve a state of equilibrium allows for not only a shorter scan duration, but is additionally amenable replacing arterial blood sampling with the much less invasive and technically demanding venous sampling to measure metabolite-corrected *C*_P,90−120_. Ultimately, this protocol improves safety and tolerability in high-risk or difficult to recruit populations, making [^18^F]SynVesT-1 PET more accessible and increasing the feasibility of larger-scale, multi-site studies.

## Electronic supplementary material

Below is the link to the electronic supplementary material.

Supplementary Material 1

## Data Availability

Data is available upon reasonable request to the corresponding or senior authors.
