# Supplementary material for: Venous blood sampling for less invasive in vivo quantification of synaptic density with constant infusion of [18F]SynVesT-1 and PET
Source: EJNMMI Res. 2025 Feb 3;15:8. doi: 10.1186/s13550-025-01200-2 (PMC11790547; doi:10.1186/s13550-025-01200-2)
Supplement: Supplementary file 1 — Supplementary Material 1 [file 13550_2025_1200_MOESM1_ESM.docx]

We thank the reviewers for their thoughtful comments. Please see below for point-by-point responses. Changes to the Manuscript have been highlighted by the use of red text in the provided “Manuscript with Marked Changes”.

**REVIEWER 1**

1: *Usually, the agreement between methods of analysis is better statistically analyzed/presented using Interclass Correlation Coefficients (ICC) and Bland-Alman plots. Please add ICCs.*

Response: We have added the intraclass correlation coefficient for equilibrium metabolite-corrected plasma concentration and *V*_T_ values to Table 1:

**Table 1.** Mean and variance of *C*_P,90-120_ and brain region *V*_T_ values, and agreement between arterial and venous-derived values (*n*=19).

|  | Arterial | | Venous | | ICC | Bias (%) |
| --- | --- | --- | --- | --- | --- | --- |
|  | Mean(SD) | COV | Mean(SD) | COV |  | Mean(SD) |
| *C*_P_,_90-120_ (SUV) | 0.32(0.07) | 20.92% | 0.28(0.07) | 24.15% | 0.92 | -12.27(10.90)% |
| Frontal Ctx. | 20.99(4.28) | 20.49% | 24.08(6.11) | 25.39% | 0.89 | 15.06(14.48)% |
| Parietal Ctx. | 20.58(4.08) | 19.80% | 23.66(5.62) | 23.74% | 0.88 |  |
| Temporal Ctx. | 21.89(4.69) | 21.42% | 25.19(6.59) | 26.15% | 0.89 |  |
| Occipital Ctx. | 20.71(4.08) | 19.69% | 23.79(5.61) | 23.58% | 0.88 |  |
| Hippocampus | 14.43(3.23) | 22.56% | 16.54(4.68) | 28.31% | 0.90 |  |
| Amygdala | 18.30(4.35) | 23.79% | 21.08(6.12) | 29.03% | 0.91 |  |
| Thalamus | 15.55(3.40) | 21.85% | 17.90(4.76) | 26.61% | 0.90 |  |
| Caudate | 18.73(4.51) | 24.07% | 21.60(6.42) | 29.69% | 0.91 |  |
| Putamen | 21.96(4.75) | 21.62% | 25.28(6.62) | 26.17% | 0.90 |  |
| Cerebellum | 16.12(3.15) | 19.55% | 18.52(4.34) | 23.45% | 0.88 |  |
| C. Semiovale | 3.35(0.60) | 18.02% | 3.81(0.56) | 14.67% | 0.83 |  |
| WB GM | 19.59(3.97) | 20.26% | 22.48(5.73) | 25.49% | 0.88 |  |

Abbreviations: equilibrium metabolite-corrected plasma concentration (*C*_P,90-120_), volume of distribution (*V*_T_), standardized uptake value (SUV), standard deviation (SD), coefficient of variance (COV), intraclass correlation coefficient (ICC), cortex (Ctx), centrum semiovale (C. semiovale), whole brain gray matter mask (WB GM).

2: *Could the authors clarify how many of their psychiatric patients were meeting criteria for a current mood disorder? How do the authors believe that the fact that not all patients met the criteria for depression at the time of inclusion in the study may influence their findings of no differences between input functions or Vt values? Please identify and discuss the patients who met the criteria for current depression in the graphs (Figure 4b).*

Response: All study participants completed Structured Clinical Interview for DSM Disorders (SCID). Of all the study participants with a lifetime mood disorder diagnosis (*n*=10), seven met criteria for current depressive episode. The remaining three did not meet criteria for current depressive episode, which was further validated by Montgomery-Asberg Depression Rating Scale (MADRS) scores less than 10, a suggested cutoff for depressive symptom remission. This information is now included with the “Human subjects” information and the individuals in symptom remission are indicated by shaded symbols in Figure 4b.

“Data were analyzed from 19 participants (10 male; 55.6±10.1 years), including nine healthy adults and ten adults with lifetime history of mood disorder diagnosis, seven of whom were currently experiencing a major depressive episode, with the remaining three being in remission from depressive symptoms as determined by the Structured Clinical Interview for DSM Disorders.”

3: *Could the authors clarify which vein was used for venous sampling in their study? Given that different venous sites might have slightly varying tracer concentration kinetics, this information is important in terms of reproducibility. How do authors believe that using a different venous sampling site might impact the assumptions underlying the venous input function?*

Response: Catheter placement has been clarified:

“Radial artery and venous (at the antecubital area) catheters were placed in the arm contralateral to the venous catheter used for administration of [^18^F]SynVesT-1.”

Catheter placement was consistent across study participants and should not have contributed to variability in the arterial versus venous measurement bias. Further, these placements were selected so as to minimize distance/time between sampling sites and limit differences between arterial and venous-derived measurements.

4: *Do the authors expect differences in protein binding between venous and arterial samples? Please discuss, especially in the context of medications in patients vs controls*

Response: Unfortunately, for these studies, protein binding (free fraction, *f*p) was only measured in the arterial plasma. This study limitation is now noted in the Discussion:

“Radiotracer protein binding (plasma free fraction, *f*p) was only measured in arterial samples. We did not observe differences in arterial *f*p as a function of diagnosis or medication status (Figure S3), but we are not able to comment on potential differential impact of diagnosis, medication status, or other factors on venous *f*p, and will therefore need to be evaluated in the future.”

**Figure S3.** There is no effect impact of diagnosis (left, *p*=0.29) or medication status (right, *p*=0.59) on arterial plasma free fraction (*f*p).

5: *Could you please clarify the methodology used to delineate the centrum semiovale in your study? While I noticed that your previous publications (Rossano 2020 and Finnema 2016) reference the use of an AAL_CS template and manual delineation, they do not provide detailed anatomical parameters or criteria for defining this region of interest. What specific anatomical landmarks or templates did you use to accurately locate the centrum semiovale in your analysis?*

Response:

The precise method for defining the centrum semiovale reference ROI can be found in Rossano 2020. This detailed information is now provided in the Supplement:

“A time activity curve was generated for a 2 mL volume of white matter within the centrum semiovale (CS) as previously described by Rossano, et al. (2020). To summarize: Individual subject MRI anatomical scans from 17 female and 30 male healthy subjects underwent probability mapping of tissue class using SPM12. To detect the central white matter (WM) regions, far from gray matter (to minimize spill-in effect), the WM probability map was smoothed with a 10 mm full-width-half-max Gaussian filter and then masked by an AAL-template CS ROI in the subject space. Finally, within the masked map, voxels with the highest probability values resulting in a 2 mL volume ROI were identified. The final CS ROI was created in AAL template space using the average of the 2 mL CS ROIs from the 47 subjects registered to the template space.”

6: *The authors evaluated the quality of achieved equilibrium in arterial and venous input functions as the slope of the line fit between 90 and 120 minutes (SUV/hour). It would also be interesting to evaluate the time stability of Ct/cp_art and Ct/Cp_ven for other ROIs as well.*

Response: Thank you for this suggestion. We now discuss the time-stability of the equilibrium *V*_T_ measures and *C*_T_/*C*_P_ ratio slopes in the text and provide a figure/table in the Supplement:

“Similarly, slopes of regional arterial *C*_T_/*C*_P_,_90-120_ were positive on average (whole brain GM: 0.86±2.08 *C*_T_/*C*_P_ per hour), with a mean absolute magnitude of 1.53±1.53 *C*_T_/*C*_P_ per hour ratios (Figure S1). Slopes for venous *C*_T_/*C*_P_,_90-120_ ratios were of a similar absolute magnitude (whole brain GM: 1.86±1.17 *C*_T_/*C*_P_ per hour) but were negative on average (-0.90±2.12 *C*_T_/*C*_P_ per hour).”

7: *Do authors have any relevant information pertaining to the outlier participant? Was this person a healthy volunteer or a psychiatric patient? Is there anything that can explain the obtained results?*

Response: The outlier participant (the individual who was removed from the plasma concentration slope analysis) was a 55 year-old male with a diagnosis of bipolar disorder 2. This individual was on a number of medications (lamotrigine 350 mg; buspirone 45mg; citalopram 10mg; trazodone 25 mg, irbesartan 75 mg) but no antipsychotics or mood stabilizers.

8: *For figure 3. B where the authors represent VTart and VTvein (n=19 individuals; n=10 ROIs), can the authors identify the different ROIs (for example by using different symbols) to provide clearer information to the reader.*

Response: This is an excellent suggestion! Figure 3B has been updated, now providing unique symbols for each ROI. As noted in our response to Reviewer 2 (comment 5), it can now be seen that subcortical structures and cerebellum consistently fall along the line of identity, and it is cortical structures and hippocampus that are more prone to *V*_T,vein_ overestimation of *V,*_Tart_.

9: *Figure 4. The Influence of (a) sex or (b) presence of a psychiatric diagnosis. Are Brain Vt values representing whole brain? Were individual ROIs evaluated? Please add and discuss.*

Response: It is our understanding that this Reviewer is asking if data presented in Figure 4 (that is, *V*_T_ bias as a function of sex or psychiatric diagnosis) was evaluated at the whole brain level and/or by individual ROI’s. Since the %bias for an individual subject is constant across all brain regions, data in Figure 4 can be thought of as representing whole brain and individual ROIs (also, see Table 1).

10: *Were the psychiatric patients included in the study taking any medication? How do authors consider that psychiatric medication should be considered in a study using venous input function? In light of the potential impact of hepatic enzyme inhibitors (e.g., fluoxetine, paroxetine, fluvoxamine), or other medical conditions on drug metabolism, how do authors consider these factors can influence arterial or venous blood samples by differentially affecting the input functions? In those cases, using WM as reference region might mitigate this potential differences in tracer clearence between groups. Please assess and discuss*.

Response: The subjects included in these analyses were recruited for an ongoing study being conducted in an older adult population. As such, 3 out of the 9 control subjects were taking medications (beyond OTCs), including psychoactive medications (gabapentin, venlafaxine, paroxetine, semaglutide). Additionally, 6 out of 10 participants in the psychiatric diagnosis group were taking psychoactive medications. We do believe that medication status is something that should be considered as a covariate in any analysis, regardless of outcome measure, whether that be *V*_T,art_, *V*_T,vein_, or *BP*_ND_, since depression and other psychiatric disorders are associated with white matter atrophy that may improve with pharmacotherapy and symptom reduction. That being said, we do not see a significant effect of medication on the bias between *C*_P,art_ vs *C*_P,vein_ at equilibrium.

We now include the following in the details in the Methods and Materials ->Human subjects section:

“These subjects were recruited for ongoing studies in an older adult population. As such, three out of the nine healthy participants were taking prescription medications, including psychoactive medications (gabapentin, venlafaxine, paroxetine, semaglutide). Additionally, six of ten participants with lifetime history of mood disorder were taking psychoactive medications.”

Finally, we have added the following to the discussion for readers to consider:

“This study is not without limitations. First worth noting is the heterogeneity of the sample population, particularly the inclusion of individuals taking medications with the potential of impacting tracer binding, metabolism, and clearance. However, by including these individuals this sample is arguable more representative of the population at large, making the results of these analyses more generalizable. While we did not observe a significant effect of medication status on the *C*_P,art_ vs *C*_P,vein_ bias at equilibrium (Figure S2), in the future, it is advisable to continue to examine and covary for the potential impact of medication status on desired outcome measures.”

**Figure S2.** There is non-significant effect of medication status on the *C*_P,art_ versus *C*_P,vein_ %bias.

12: *Could authors show a comparison of VTs between study groups (healthy controls vs patients with lifetime history of mood disorders) for the White Matter (Centrum Semiovale) using both venous and arterial samples?*

Response: We provide this analysis, here, to satisfy this Reviewer’s request: however, we do not include this analysis in the manuscript, as we are not powered to make any conclusive statements and correct for covariates such as medication, sex, age, etc., and use of the centrum semiovale as a reference region is not the focus of this manuscript.

A repeated measures ANOVA indicates there is a significant difference between *V*_T,art_ and *V*_T,vein_, as there is for all ROIs (F=19.51, *p*=0.0004) and a non-significant effect of diagnosis (F=12.85, p=0.0740).

13: *For purposes of reproducibility, please provide more details: a) injection pump used, b) syringe volume and materials, c) was the bolus manual, with the same syringe pump, or with a secondary syringe pump? d) bolus duration. Please discuss pitfalls and solutions.*

Response: Details regarding the automated bolus-plus-infusion protocol have been added to the Methods and Materials section. Further, for the purpose of reproducibility, individuals requiring further information about specific on the syringe and the pump program code, etc., are encouraged to contact the corresponding and senior authors.

“Doses of 163.4±27.0 MBq with high molar activity (142.0±83.9 GBq/µmol) were delivered as bolus (1 minute) followed by a constant infusion (*K*_bol_=150 minutes) using an automated infusion pump program (PHD 22/2000, Harvard Apparatus).”

14: *Is “input function” the right keyword, considering that there is not a compartmental kinetic model? Would be it better to speak about “arterial/venous blood samples”?*

Response: We appreciate this Reviewer’s attention to the details and preciseness regarding nomenclature. The keyword “input function” has been replaced with “arterial/venous blood sampling”, and throughout the manuscript, “input function” has been replaced with “plasma concentration (*C*_P_)” or “equilibrium metabolite-corrected plasma concentration (*C*_P,90-120_).” Further, the manuscript title has been updated:

“Venous blood sampling for less invasive in vivo quantification of synaptic density with constant infusion of [^18^F]SynVesT-1 and PET”

15: *Figure 4e, there is a significant correlation, but it is very weak. Please clarify and discuss.*

Response: The correlations presented in Figure 4e (now Figure 4f) – relationships between the quality of equilibrium achieved in the plasma and %difference between *V*_T_ – are not significant for arterial (r=-0.266, p=0.272) or venous (r=-0.263, p=0.277) *C*_P90-120_, therefore we do not discuss further.

**REVIEWER 2**

1: *Page 4 row 55 measears -> measures*

Response: Thank you, this has been fixed.

2: *In the statistical analyses: Pearson's correlation have the assumption that the variables correlated are normally distributed, if some of the variables were not so, I suggest Spearman's rho or transforming the variables to "make them normal", but it is advisable at least to inform the reader that the normality check was performed.*

Response: Thank you for bringing this to our attention. The “Statistical analysis” section has been updated.

“After conducting Shapiro-Wilk’s normality test, relationships between two continuous variables were assessed by Pearson’s or Spearman’s correlations as appropriate.”

3: Page 7 row 21 corelated -> correlated

Response: Thank you, this has been fixed.

4: Page 9 row 14 (n=19) but wasn't one subject removed from the analyses? Figure 3 shows data for 19 subjects but wasn't one subject removed from the analyses?

Response: Only for the analysis of arterial and venous plasma concentration slopes did we identify a statistical outlier. Data from the full sample (n=19) were included for all other analyses, including those referenced on Page 9 row 19 and in Figure 3. This has hopefully now been clarified in the text:

“When assessing *C*_P90-120_ slopes, one participant had arterial and venous slopes >3 SDs beyond the mean and was thus excluded from the slope analysis.”

5: *Figure 3b it looks there are two distinct population of pairs of values towards the upper end, one above and one below the solid line. Have you considered testing whether a factor among the one measured splits such regression in two? Dose (median)? Maybe the scanner? Gender? Diagnosis? I have seen some of these suggested factors evaluated for the %Difference, but I would like to see the group difference highlighted in Figure 3b as well. It can be supplementary.*

Response: In response to Reviewer 1, comment 8, Figure 3b has been updated with unique symbols for each of the 10 ROIs. It can now be seen that subcortical structures and cerebellum consistently fall along the line of identity, and it is cortical structures and hippocampus that are more prone to *V*_Tvein_ overestimation of *V*_Tart_. In the supplement (Figure S1) we include an additional variation of the graph that highlights datapoints from three subjects with the greatest deviation and provide demographic and study details for each.

**Figure S1.** The correlation between *V*_Tart_ and *V*_Tvein_ (top). Datapoints representing frontal, parietal, temporal, and occipital cortices and the hippocampus of three individuals whose data separate from the line of identity (dasher red line) are highlighted. Demographic and injection-related variables are provided for these three subjects in the table (bottom).

6: *The scanner effect could also be tested for the %Difference figures.*

Response: We have now added a panel to Figure 4 showing the (non)effect of scanner.
